# Supplementary material for: Chromothripsis during telomere crisis is independent of NHEJ, and consistent with a replicative origin
Source: Genome Res. 2019 May;29(5):737–49. doi: 10.1101/gr.240705.118 (PMC6499312; doi:10.1101/gr.240705.118)
Supplement: Supplemental Material [file supp_gr.240705.118_Supplemental_file_1.zip › contigs/annotated_contigs/DB56/contig.2.DB56_length_273_mean_cov_4.94139194139.docx]

**DB56_length_273_mean_cov_4.94139194139**

```
AGCTACATATATCAACTCATTGAATTCTCACAACAACAAAATAAGTGCTATCATTGTTACCATTGTCATTGTCACCATTATCACCATCA
 >chr6:99717289-99717430 - E=1e-64 p=0e+00
TGTGGCTCCATCATCATCACCACCATCACCATTGTCATCA|TCATCACCACCA|TCACCATTGTCACCATCACCACCACCACCACGACC
                                         >chr6:99717184-99717328 - E=2e-75
ACCACCACCATCCTCACCGTCACCATCACCATCATCATCATCATCTCAACAGATTAGAAACTGAGGCAAAGTGAGGTTACATAACTCAC

TGGAGATC
```
